# Symmetrically Erupted Upper Fourth Molars and an Impacted Fifth Molar: A Case Report

**DOI:** 10.1155/crid/3575942

**Published:** 2025-07-04

**Authors:** Jian Shi, Ye Bi, Zhi Wu

**Affiliations:** ^1^Department of Stomatology, Shenzhen Song-Gang People Hospital, Shenzhen, Guangdong, China; ^2^Department of Stomatology, Shenzhen Baoan Air-Sea Hospital, Shenzhen, Guangdong, China

**Keywords:** fifth molar, fourth molar, paramolar, supernumerary tooth

## Abstract

**Introduction:** Supernumerary teeth are often asymptomatic and impacted within the jawbone, typically identified during routine radiographic examinations. While cases of impacted fourth molars have been reported, bilaterally erupted fourth molars are exceedingly rare. This case report presents a unique instance of symmetrically erupted maxillary fourth molars, along with an impacted fifth molar in the right upper jaw.

**Case Presentation:** A 38-year-old male presented with food impaction and discomfort in the area of the left maxillary third molar. Clinical examination revealed bilaterally erupted fourth molars, and a panoramic radiograph incidentally uncovered an impacted fifth molar in the right maxilla. Cone beam computed tomography confirmed these findings. The left maxillary fourth molar was diagnosed with periapical periodontitis. Both the left maxillary third and fourth molars were extracted under local anesthesia. Periodic follow-up of the right fourth and fifth molars was recommended based on symptom progression and patient preference. The patient expressed satisfaction with the treatment plan and surgical outcome.

**Conclusion:** This report documents a rare case of symmetrically erupted fourth molars with an associated impacted fifth molar in the maxilla. The treatment strategy and surgical approach should be tailored to the patient's symptoms and willingness to undergo the recommended procedures.

## 1. Introduction

Supernumerary teeth (ST) are developmental anomalies characterized by an excess number of teeth in either the primary or permanent dentition. These additional teeth can occur unilaterally or bilaterally, as a single tooth or multiple teeth, and may be present in one or both jaws. ST are classified based on their location: mesiodens (situated between the two maxillary central incisors), paramolars (located between the second and third maxillary molars), and distomolars (positioned distal to the third molar) [1, 2].

The prevalence of ST ranges from 0.3% to 3.8% in the general population, with a slightly lower prevalence of 1.5% to 3.5% in individuals with permanent dentition. ST are more common in males, with a male-to-female ratio of approximately 2:1 [2, 3].

ST may erupt normally or remain impacted within the jawbone, often remaining asymptomatic. They are often discovered incidentally during routine imaging, such as panoramic radiography, usually prompted by dental complications like swelling, dental caries, occlusal interference, and pericoronitis or by other odontological complaints from patients at the dental clinic.

Based on searching on electronic databases of PubMed and Chinese databases (CNKI, Wanfang, and VIP databases), no similar case was found.

## 2. Case Presentation

A 38-year-old male presented to the Department of Stomatology with a chief complaint of food impaction and occasional discomfort in the region of the left maxillary third molar, persisting for several months. The patient reported no significant personal or family medical history and was generally in good health.

### 2.1. Clinical Examination

**Figure 1:**
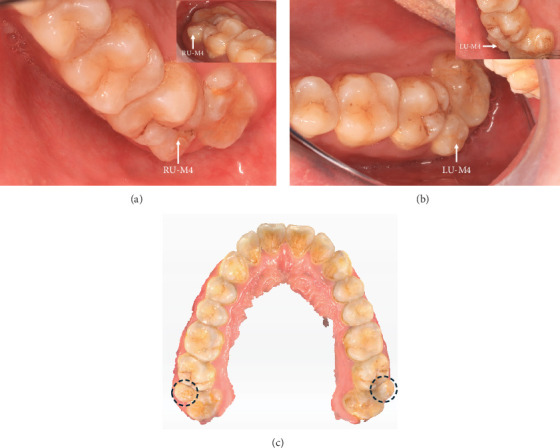
(a, b) Occlusal views of the right upper fourth molar (RU-M4) and the left upper fourth molar (LU-M4). (c) Digital scan of the occlusal aspect of the maxillary dentition, with the supernumerary fourth molars indicated by dotted circles.

Vital signs included a blood pressure of 122/78 mmHg and a pulse rate of 70 beats per minute. Intraoral examination unexpectedly revealed symmetrically positioned bilateral maxillary fourth molars in the buccal embrasures between the second and third molars (Figure 1). The third molars appeared fully formed with no apparent decay, though the patient reported discomfort during percussion. Laboratory tests revealed that the patient's complete blood count and coagulation indices were within normal reference ranges.

### 2.2. Imaging Studies

**Figure 2:**
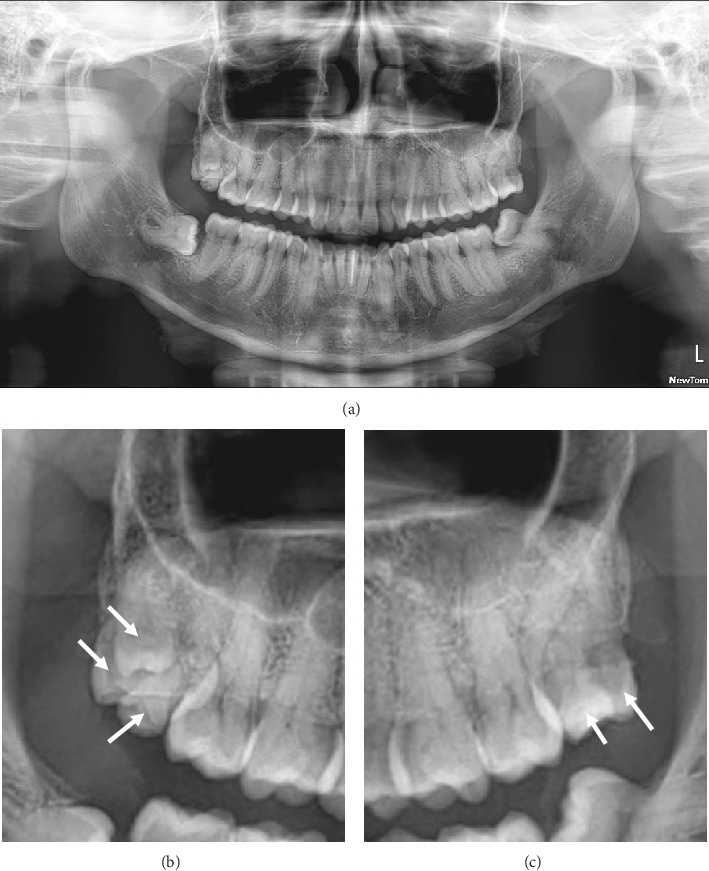
(a) Panoramic radiograph of a 38-year-old male patient showing the presence of maxillary supernumerary molars in each hemiarch. (b) Screenshotted panoramic radiograph of the right maxillary quadrant. (c) Screenshotted panoramic radiograph of the left maxillary quadrant. The white arrows point to the left third molar and the supernumerary fourth molar.

Panoramic radiography showed that the left upper third and fourth molars overlapped in the two-dimensional image (Figure 2). Notably, three additional tooth-like structures were observed in the region of the right upper third molar. The patient was unaware of these ST and had no knowledge of similar conditions in his family.

**Figure 3:**
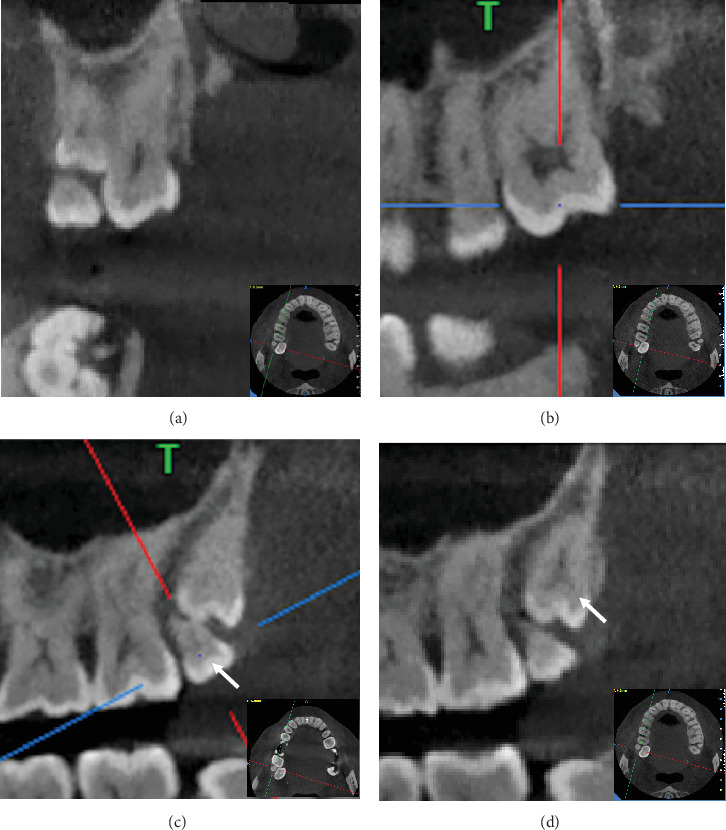
(a) Coronal view of the patient's CBCT scan showing the maxillary third, fourth, and fifth molars in the right maxillary quadrant. (b–d) Sagittal views of the maxillary (b) third, (c) fourth, and (d) fifth molars in the right maxillary quadrant. The white arrow points to the right maxillary fourth molar.

**Figure 4:**
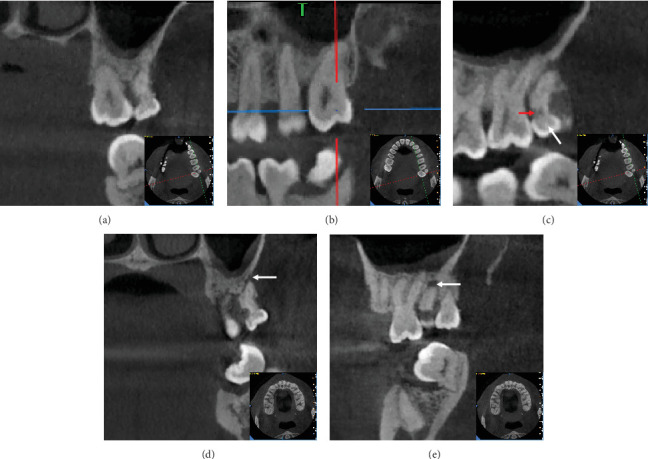
(a) Coronal view of the patient's CBCT scan showing the maxillary third and fourth molars in the left maxillary quadrant. (b, c) Sagittal views of the maxillary (b) third molar and (c) fourth molar in the left maxillary quadrant. (c) The red arrow indicates an area of decreased density on the mesial aspect of the fourth molar root, while the white arrow points to the left maxillary fourth molar. (d, e) White arrows highlight areas of decreased density in the periapical region of the fourth molar root.

To clarify the morphology, three-dimensional positioning, root structure, and relationships with adjacent anatomical structures, a cone beam computed tomography (CBCT) scan was performed (Figures 3 and 4). The CBCT confirmed the presence of an impacted fifth molar in the right upper maxilla (RU-M5). Coronal views showed the impacted fifth molar positioned buccal to the third molar, while sagittal views revealed it was distal to the fourth molar. The crowns of both the fourth and fifth molars exhibited molariform characteristics.

### 2.3. Final Diagnosis

Impacted teeth, ST, and periapical periodontitis of the left upper fourth molar (LU-M4) were diagnosed based on the clinical and radiographic examinations.

### 2.4. Treatment and Results

**Figure 5:**
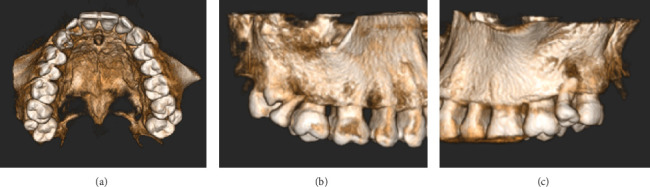
(a) Occlusal view of the maxillary dentition from a three-dimensional reconstruction of the CBCT scan. (b, c) Sagittal views of the (b) right and (c) left maxillary hemiarches.

**Figure 6:**
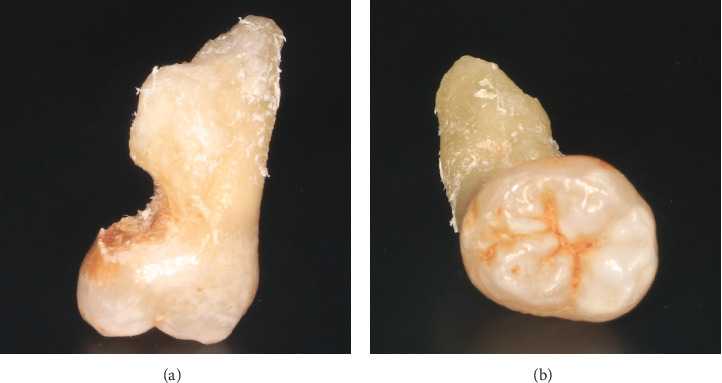
(a, b) The extracted fourth molar from the left upper maxilla.

Considering the patient's preferences and symptomatology, along with a detailed analysis of the CBCT scan and 3D reconstruction (Figure 5), it was decided to extract the left maxillary third and fourth molars. The procedure was performed under local anesthesia, with the patient in aseptic conditions. Anesthetic was infiltrated into the left upper posterior maxilla, and the extractions were completed without perioperative complications. Examination of the extracted left maxillary fourth molar revealed decay on the proximal and lingual surfaces (Figure 6). The extraction socket was curetted and irrigated with saline, and platelet-rich fibrin (PRF) prepared from the patient's blood was packed into the socket to promote healing. The surgical site was closed with 3-0 sutures, and the patient was prescribed chlorhexidine mouth rinse (0.12%) twice daily for 5 days, along with an analgesic (diclofenac sustained-release capsules, 50 mg). Sutures were removed 1 week postoperatively. The patient chose not to proceed with the preventive extraction of the right upper wisdom tooth and the supernumerary molars, as there were no symptoms present. Regular follow-up appointments were recommended. The patient reported minimal postsurgical pain, and at the 1-week follow-up, the wound exhibited normal healing.

## 3. Discussion

The etiology and pathogenesis of ST remain unclear. Proposed theories include genetic predisposition, atavism, hyperactivity of the dental lamina, and environmental factors [4, 5]. There is evidence suggesting a correlation between ST and certain hereditary conditions, such as Ehlers–Danlos syndrome, Gardner syndrome, and cleidocranial dysplasia [6]. Notably, the patient in this case did not present with any of these conditions. Variants in the *FER1L6* and *PDGFRB* genes have been linked to nonsyndromic ST [7, 8]. Recent studies have also identified a connection between heterozygous variants in *FREM2* and the presence of mesiodens and ST [9]. These findings highlight the importance of retaining blood samples for further genetic testing and research in future cases of ST, particularly paramolars.

ST can be classified by shape into conical, tuberculate, molariform, and supplemental types [10, 11]. The conical supernumerary tooth, often peg-shaped, is the most common in permanent dentition and typically appears as a mesiodens, which may cause rotation or displacement of the permanent incisor but rarely delays eruption. Tuberculate STs, characterized by one or more cusps or tubercles and delayed root formation, are often found on the palatal side of the central incisor and are associated with delayed incisor eruption. Supplemental STs are duplications of teeth at the end of a tooth series, with the permanent maxillary lateral incisor being the most common, followed by supplemental premolars and molars [12]. The bilateral maxillary fourth molars (paramolars) and the impacted fifth molar in this case are molariform in shape and smaller than typical molars, though STs are often rudimentary in shape.

A review of the literature revealed only three original case reports [13–15] in English regarding fifth molars, based on searches of the PubMed database complemented by additional manual efforts. Searches conducted within Chinese databases (CNKI, Wanfang, and VIP databases) yielded no cases of supernumerary fifth molars. The rarity of fifth molars is evident. The details of the reported cases are summarized in Table 1. All reported patients were female, aged 20 to 33 years. Among the five impacted ST, one was impacted in soft tissue. Two cases [13, 15] exhibited symptoms unrelated to ST, while one case [14] reported pressure in the maxillary third molar region bilaterally. Two cases underwent preventive removal [13, 14], and one was recommended for periodic follow-up [15]. Due to the limited number of published cases, no definitive conclusions can be drawn about the prevalence of fifth molars by sex.

ST are typically impacted and asymptomatic, often discovered incidentally during panoramic or CBCT imaging, and may remain impacted throughout an individual's life. However, complications can arise from impacted STs, including tooth decay, root resorption of adjacent teeth, periapical periodontitis, and dentigerous cysts. Prophylactic extraction of ST is recommended to prevent such complications, especially in symptomatic cases. CBCT is an essential tool for accurate preoperative assessment and surgical planning. For asymptomatic patients who are hesitant to undergo surgery or are at higher surgical risk, periodic follow-up and monitoring should be considered.

## 4. Conclusion

ST are frequently impacted, and the occurrence of a fifth molar is exceptionally rare. This case report details a unique instance of symmetrically erupted fourth molars on both sides of the maxilla, along with a hidden fifth molar on the right. Treatment should be guided by the patient's symptoms and preferences. Additionally, reporting of similar cases is encouraged to enhance the understanding of supernumerary molars.

## Figures and Tables

**Table 1:** General characteristics of studies on the maxillary fifth molar.

**Study**	**Type**	**Age of patient**	**Gender**	**Laterality**	**State**	**Symptoms/coexisting problems**	**Management**
Kokten et al., [13]	Case report	20	Female	Unilateral	Impacted	Temporomandibular joint disease	Preventive removal
Asrani et al., [15]	Case report	33	Female	Bilateral	Impacted (one in soft tissue)	Bleeding gums around fixed dentures	None and periodic follow-up
Husain et al., [14]	Case report	29	Female	Bilateral	Impacted	Severe tenderness in the region of the maxillary third molars on both sides/localized gingivitis in the left maxillary region	Removal

## Data Availability

The data that support the findings of this study are available from the corresponding author upon reasonable request.
